# Climatic Suitability from MaxEnt Models Reflects Growth Performance in European Forest Trees

**DOI:** 10.3390/plants15081140

**Published:** 2026-04-08

**Authors:** Ricardo Enrique Hernández-Lambraño, José Ángel Sánchez-Agudo

**Affiliations:** 1 Department of Forestry Engineering, Silviculture Laboratory, Dendrochronology and Climate Change, DendrodatLab-ERSAF, University of Cordoba, Campus de Rabanales, Crta. IV, km. 396, 14001 Córdoba, Spain; enriquericardo.hl@gmail.com; 2 Department of Botany and Plant Physiology, Botany Area, University of Salamanca, Miguel de Unamuno Campus, Licenciado Méndez Nieto s/n, 37007 Salamanca, Spain

**Keywords:** species distribution models, climatic suitability, dendrochronology, tree growth, climate variability, forest vulnerability

## Abstract

Species distribution models (SDMs) are widely used to define climatic constraints on species ranges, yet their ability to reflect demographic processes remains poorly understood. We integrated annually calibrated SDMs (1981–2005) with tree-ring width data from 15 European forest species in the Iberian Peninsula to evaluate if climatic suitability mirrors tree growth, particularly for populations at their climatic tolerance limits. Our results show that higher suitability consistently relates to reduced growth decline, acting as a reliable proxy for forest vigor. Notably, interannual variability in climatic suitability was positively associated with growth, suggesting that climatic fluctuations may enhance physiological resilience. We also found that Mediterranean species exhibit higher growth sensitivity to climatic suitability changes than Eurosiberian species. These findings demonstrate that SDMs can capture functional constraints beyond mere presence, positioning annual climatic suitability as a key predictor of radial growth and offering valuable insights for forest management under climate change.

## 1. Introduction

Climate is widely recognized as a primary abiotic determinant of species distributions at broad spatial scales, although its influence may be modulated by biotic interactions, edaphic conditions, and microenvironmental heterogeneity at local scales. Despite this, the mechanisms linking climatic conditions to demographic processes that control species’ persistence remain one of the major challenges in contemporary ecology [1]. Species Distribution Models (SDMs) have enabled the quantitative characterization of climatic suitability gradients and the projection of potential distributional ranges under scenarios of global change [2]. However, the implicit assumption that higher climatic suitability translates directly into higher species performance has rarely been consistently supported by empirical evidence [1,3].

This lack of correspondence has raised a central question in the ecological interpretation of SDMs: to what extent do SDM-based climatic suitability gradients reflect the capacity of populations to maintain growth and persistence across contrasting climatic conditions?

A key conceptual advance was provided by [4], who demonstrated that climatic gradients shape not only the magnitude but also the architecture of demographic strategies [5]. Nevertheless, multiple processes may modify or even decouple this relationship, leading to weak or non-linear responses between climate and population performance [6,7]. Intense biotic interactions, the availability of favorable microhabitats, demographic buffering, or high individual longevity can allow populations to persist in climates that appear suboptimal based on broad-scale climatic suitability alone [8,9]. In addition, compensatory demographic responses and density-dependent processes may stabilize populations across wide environmental gradients [10].

However, these buffering mechanisms can be constrained or eroded by temporal climatic variability, a frequently underestimated component that fundamentally alters the relationship between climatic suitability and population performance. In regions experiencing strong interannual fluctuations, such as rear-edge portions of species distributions, populations are exposed to alternating sequences of favorable and unfavorable years that non-linearly affect survival, growth, and reproduction [11,12]. Such variability not only amplifies thermal and hydric stress, but also generates lagged and cumulative effects that can reshape age structure and reduce recovery capacity following extreme events [13]. Consequently, discrepancies between climatic suitability projected by SDMs and observed population performance may reflect the modulatory influence of ecological history, physiological memory, and recurrent exposure to high climatic variability [14].

Among the various drivers of forest dynamics, climatic variability frequently contributes to gradual reductions in individual growth—a sensitive indicator of tree performance—which, together with other factors such as competition and site conditions, may precede detectable demographic decline [15]. Despite its central relevance, spatially and temporally replicated growth data suitable for testing suitability–performance relationships across broad climatic gradients remain remarkably scarce, limiting empirical assessments of whether SDM-derived gradients translate into functional constraints on forest performance. Accordingly, integrating SDM-derived climatic suitability projections with empirical measures of species performance represents a critical step towards a mechanistic understanding of species vulnerability to climate change. This integrative framework not only allows testing whether suitability gradients coherently predict spatial variation in growth, but also enables the identification of the vital-rate mechanisms underpinning local persistence [16,17]. As argued by Ehrlén and Morris [18], only by synthesizing niche modelling with environmentally driven demography can the descriptive concept of “suitability” be transformed into a process-based measure of ecological performance. Ultimately, such integration is essential for anticipating climate-change impacts on forest population regeneration and resilience, providing a robust empirical basis for linking large-scale distributional patterns with the demographic mechanisms that sustain them.

Despite the widespread use of SDMs to estimate climatic suitability, a key unresolved question is whether these estimates reflect meaningful differences in species performance across environmental gradients. While several studies have attempted to link SDM outputs with demographic rates or population dynamics [4,5,16], empirical evidence remains limited, particularly for long-lived organisms such as forest trees. As a result, the ecological interpretation of SDM-derived suitability remains debated, and its relationship with functional indicators of population performance is still poorly understood.

Here, we assess whether climatic suitability gradients derived from SDMs are associated with spatial patterns of growth reduction in forest tree species across the Iberian Peninsula. We integrate an extensive dendrochronological dataset—comprising annually resolved radial growth series from 15 tree species sampled along broad climatic gradients—with independent estimates of climatic suitability obtained from SDMs calibrated using continental-scale occurrence data. This approach allows us to test whether populations occurring under climatically less suitable conditions exhibit reduced growth, a sensitive indicator of physiological stress and demographic performance in long-lived organisms.

By focusing on individual growth responses, our study explicitly links spatial variation in climatic suitability to underlying demographic processes, providing a functional evaluation of SDM predictions under heterogeneous climatic conditions. Within this framework, we hypothesize that (i) tree growth declines towards areas of lower climatic suitability, (ii) this relationship is amplified under higher climatic variability, and (iii) species differ in their growth responses along suitability gradients according to their biogeographic affinities, with Eurosiberian taxa showing stronger growth reductions than Mediterranean species.

## 2. Materials and Methods

### 2.1. Study Area and Species

**Figure 1:**
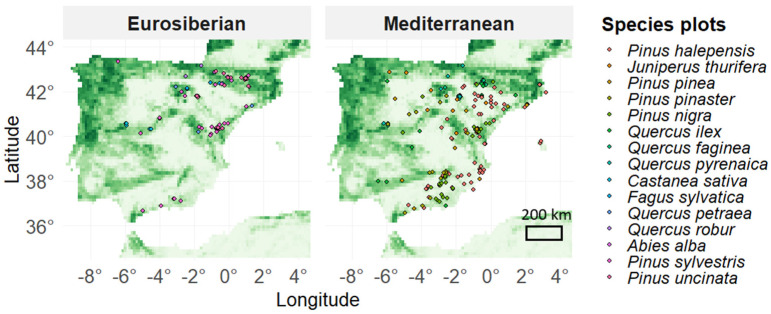
Spatial distribution of the tree-ring width network (RWI−net; Gazol et al., [21]). Sampling sites are categorized by taxon and biogeographic affinity (Eurosiberian vs. Mediterranean). The base map indicates forest cover density (percentage of area covered by trees is shown in green) within the study area.

The study area encompasses the Iberian Peninsula, a biogeographically distinctive region that functions as a major transition zone between temperate Eurosiberian and Mediterranean biomes in southwestern Europe (Figure 1). Its geographical configuration—bounded by the Pyrenees to the north and the Strait of Gibraltar to the south—together with pronounced altitudinal heterogeneity, gives rise to sharp climatic, edaphic, and ecological gradients [19]. From a biogeographical standpoint, the Iberian Peninsula constitutes a complex mosaic of Pleistocene glacial refugia and contemporary range limits for many European forest taxa. Northern and central mountain systems delineate the southern distributional margins of several Eurosiberian species, whereas Mediterranean taxa dominate lowland and coastal areas in the southern and eastern sectors [20,21]. This spatial convergence of Eurosiberian and Mediterranean lineages, coupled with strong gradients in temperature and water availability, makes the Iberian Peninsula an especially suitable natural laboratory to examine how climatic suitability modulates forest growth responses under increasing aridity and recurrent drought stress [22].

We analyzed fifteen European forest species representing two major biogeographical groups: Eurosiberian and Mediterranean species. The Eurosiberian group comprised *Fagus sylvatica* L., *Quercus robur* L., *Q. petraea* (Matt.), Liebl., *Pinus sylvestris* L., *Abies alba* Mill., *Castanea sativa* Mill., and *P. uncinata* Ramond ex A.DC. These species are characteristic of temperate to boreal or montane forests and are widely distributed across central and northern Europe, with many populations reaching their rear-edge limits in the Iberian Peninsula. They typically exhibit a strong dependence on relatively cool temperatures and high water availability, and their growth performance is known to be particularly sensitive to drought and heat stress near the southern margins of their ranges [23,24,25,26]. The Mediterranean group included also sclerophyllous and marcescent oak species (*Q. ilex* L., *Q. faginea* Lam., *Q. pyrenaica* Willd.) together with Mediterranean conifers (*P. halepensis* Mill., *P. nigra* J.F.Arnold., *P. pinea* L., and *Juniperus thurifera* L.), whose distributions are largely centered in the circum-Mediterranean region. These species are adapted to climates characterized by strong seasonal water deficits, hot and dry summers, and high interannual climatic variability. They display a set of functional traits that enhance drought resistance and resilience, including tough evergreen foliage, conservative hydraulic strategies, deep or extensive root systems, and high physiological plasticity [20,27,28,29]. Nevertheless, substantial functional and ecological heterogeneity exists within this group, with some species occupying transitional environments between temperate and Mediterranean climates (e.g., *Q. faginea*, *Q. pyrenaica*) or cold and dry continental plateaus and montane systems (e.g., *P. nigra*, *J. thurifera*), highlighting the importance of accounting for biogeographical context when evaluating growth responses to climatic suitability.

### 2.2. Species and Climatic Data

**Figure A1:**
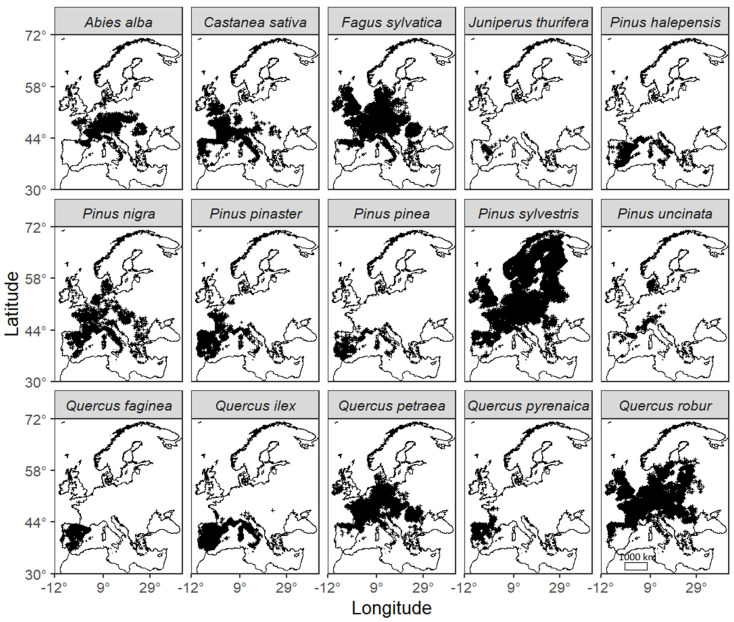
Geographical distribution of presence used in model calibration.

To characterize the distribution of the selected forest tree species (Figure A1), we sourced presence data at a 1 × 1 km spatial resolution from the EU-Forest dataset [30]. This database consolidates information from approximately 250,000 National Forest Inventory plots across the majority of European countries, encompassing 588,983 occurrence records for 242 tree species. To mitigate spatial autocorrelation and prevent model overfitting [31], we applied spatial thinning with a 5 km minimum distance threshold using the ntbox package [32] in R [33]. The tree species included in this study were selected based on the availability of high-quality dendrochronological series with sufficient temporal coverage and spatial replication within the study area. These species collectively represent the main biogeographic groups occurring in the Iberian Peninsula, including both Mediterranean and Eurosiberian taxa, allowing us to evaluate whether the relationship between climatic suitability and growth performance is consistent across contrasting ecological strategies.

Climatic variables were utilized to estimate species-specific climatic suitability across their respective European ranges. High-resolution (1 km^2^) climate data were retrieved from the CHELSA v2.1 global database [34]. Based on monthly layers of precipitation, maximum temperature, and potential evapotranspiration, we calculated mean values specific to the growing season—defined here as the period from April to September—for each year within the 1981–2005 study interval [35]. These predictors were selected due to their robust physiological link to tree growth, as well as their influence on cumulative drought stress and broader distributional patterns [21,36]. By aligning the climatic suitability metrics with the specific timeframe and phenological window of growth, we ensured a biologically relevant coupling between environmental filtering and forest productivity.

### 2.3. Climatic Suitability Modeling

To estimate the climatic suitability of the focal species, we employed the Maximum Entropy algorithm (MaxEnt; [37]), a method widely recognized for its robustness in characterizing species–environment relationships and its ability to capture complex, non-linear interactions among predictors [2,38].

To capture temporal dynamics in climatic suitability, we followed a two-step projection approach. First, a master SDM was calibrated for each species using all occurrence records and long-term climate averages to establish stable niche parameters (i.e., the training data remained constant to define the species’ climatic profile). Second, these pre-established model parameters were projected onto annual climatic layers for the 1981–2005 period. By keeping the model coefficients constant and varying only the environmental predictors annually, we ensured that the resulting interannual suitability values specifically reflect the response of the species to year-to-year climatic fluctuations, avoiding the noise that would arise from re-estimating parameters annually. SDMs were calibrated using climatic predictors describing temperature and water availability, including annual precipitation, mean maximum temperature and potential evapotranspiration. These variables were selected because they capture key climatic constraints on tree growth in Mediterranean and temperate ecosystems [21]. Prior to model calibration, multicollinearity among predictors was evaluated using pairwise Pearson correlation coefficients, and highly correlated variables (|r| > 0.7) were not included simultaneously in the models.

To optimize model complexity and mitigate overfitting, we implemented a rigorous tuning procedure using the gridSearch function from the SDMtune package in R [39], following the framework proposed by [40]. We evaluated 12 candidate models per species, exploring a range of regularization multipliers (0.5, 1, 1.5, and 2) and three feature class combinations (Linear, L; Quadratic, Q; and the interaction LQ). The calibration area (M) was delimited by a Minimum Convex Polygon (MCP) encompassing the entire European range of each species (Figure A1). This broad geographic extent captures the full realized niche—from the northern temperate core to the southern Mediterranean rear-edges—ensuring that the model encompasses the complete environmental gradient and adaptive variability [41,42]. Within each MCP, we randomly generated 10,000 background points to adequately sample the available environmental space. Model selection followed a stringent sequential procedure. For each species, candidate models were first filtered based on the corrected Akaike Information Criterion (AICc), retaining only those with a delta AICc < 2 to ensure parsimony [43]. From this subset of top-performing models, we selected the final model that yielded the highest Area Under the Receiver Operating Characteristic Curve (AUC*test*) using a 70/30 split for training and internal validation [44]. This two-step selection process prioritizes models that are both information-theoretically sound and highly predictive of independent data. AUC was used as a general measure of model discrimination ability to ensure that SDMs achieved acceptable predictive performance prior to extracting climatic suitability estimates for subsequent analyses.

The selected optimal models were projected onto annual climatic layers for the 1981–2005 period across the study area. We retained the raw MaxEnt output as it provides a continuous metric of suitability that is ecologically interpretable as a measure of environmental quality [45]. Finally, we quantified the relative importance of environmental predictors through permutation importance and analyzed response curves to identify the climatic thresholds and ranges that govern climatic suitability, and by extension, the climatic potential for growth across the European forest network.

### 2.4. Tree Growth Data

To characterize growth responses across the study period, we used growth reduction data from a previously published dataset by Gazol et al., [21] (Figure 1; Table A1). This dataset is based on a comprehensive network of tree-ring width chronologies across the Iberian Peninsula for the period 1981–2005. In this framework, growth reduction was quantified as the negative deviation of the observed annual Ring Width Index (RWI*obs*) relative to the long-term mean growth (RWI*mean*). By leveraging this established dataset, we aimed to evaluate the efficacy of SDM-derived climatic suitability as a diagnostic tool for forest performance. Specifically, we used these growth reduction indices as a benchmark to assess whether interannual fluctuations in climatic suitability effectively capture the declines in tree growth for 15 European forest species. This approach allows for a robust validation of suitability metrics as a proxy for the ecological and physiological constraints shaping forest growth at a continental scale.

### 2.5. Statistical Analysis

To quantify the relationship between SDM-derived climatic suitability and tree growth, we fitted linear mixed-effects models (LMMs). This framework allowed us to explicitly test whether spatial and temporal gradients in climatic suitability are associated with observed patterns of growth reduction across the Iberian Peninsula. The growth reduction index was used as the response variable. As fixed effects, we included mean annual climatic suitability and its temporal variability, expressed as the standard deviation, to evaluate whether growth declines are stronger under climatically marginal conditions and whether this effect is intensified by increased climatic variability. To explicitly address differences among species with contrasting biogeographic affinities, we included biogeographic distribution type (Eurosiberian vs. Mediterranean) as an additional categorical fixed effect. This term allows us to test whether species groups differ systematically in their growth sensitivity to climatic marginality. Moreover, by including interactions between distribution type and both climatic suitability and its variability, we assessed whether the magnitude and direction of suitability–growth relationships depend on biogeographic affiliation.

To accommodate the hierarchical structure of the data and account for both taxonomic and spatial sources of non-independence, we included species identity and site as random intercepts in the mixed-effects models. This random-effects structure controls for repeated observations within the same species and for shared environmental and historical conditions among trees sampled at the same site, while allowing baseline growth levels to vary across taxa and locations [4]. Model assumptions were evaluated by inspecting residual distributions and residual-versus-fitted plots to assess normality and homoscedasticity.

By integrating long-term dendrochronological series from 15 tree species with independent, continental-scale estimates of climatic suitability, our modeling framework tests whether populations occurring in climatically marginal environments exhibit reduced radial growth, a sensitive proxy of demographic performance in long-lived organisms. This design allows us to assess whether SDM-derived suitability metrics reflect biologically meaningful constraints on growth under variable climatic conditions, while explicitly quantifying differences between Eurosiberian and Mediterranean species according to their biogeographic affinities. Statistical analyses were performed using linear mixed-effects models implemented with the “lmer” function from the lmerTest package [46] in the R environment [33].

To improve grammatical accuracy and clarity, text and image elements were reviewed with assistance from ChatGPT-4o (OpenAI, 2026, San Francisco, CA, USA). The tool was used solely for grammatical revision and minor layout adjustments. All content was subsequently critically reviewed, edited, and finalized by the authors to ensure accuracy and coherence.

## 3. Results

### 3.1. Habitat Suitability Modelling

SDM performance ranged from moderate to good across the fifteen species. Over the 1981–2005 period, mean AUC*test* values for the best models varied between 0.66 and 0.90, with some species approaching 0.82 and others remaining near 0.66.

Across species, evapotranspiration was generally the main driver of climatic suitability, although the dominant predictor varied by species and biogeographic group (Table A3). Mediterranean species showed a more balanced contribution of hydric and thermal variables, while Eurosiberian species often depended on a single dominant climatic factor. For instance, maximum temperature was most influential for *P. uncinata* and *F. sylvatica*, whereas precipitation dominated for *A. alba*.

**Figure 2:**
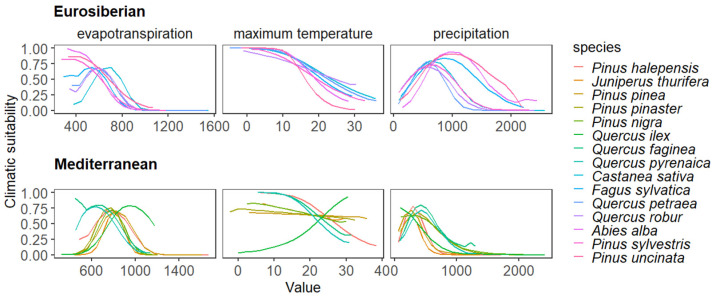
Response curves for Eurosiberian and Mediterranean tree species showing the functional relationship between climatic suitability predicted by MaxEnt models and each climatic predictor.

Climatic response curves revealed clear environmental partitioning between biogeographic groups (Figure 2). Eurosiberian species displayed narrow, unimodal niches at the colder and wetter end of the Iberian climate spectrum, with high sensitivity to increases in temperature or reductions in precipitation. Mediterranean species exhibited broader and more variable niches, shifted towards warmer and drier conditions, with optima at higher evapotranspiration and more flexible temperature and precipitation ranges.

**Figure 3:**
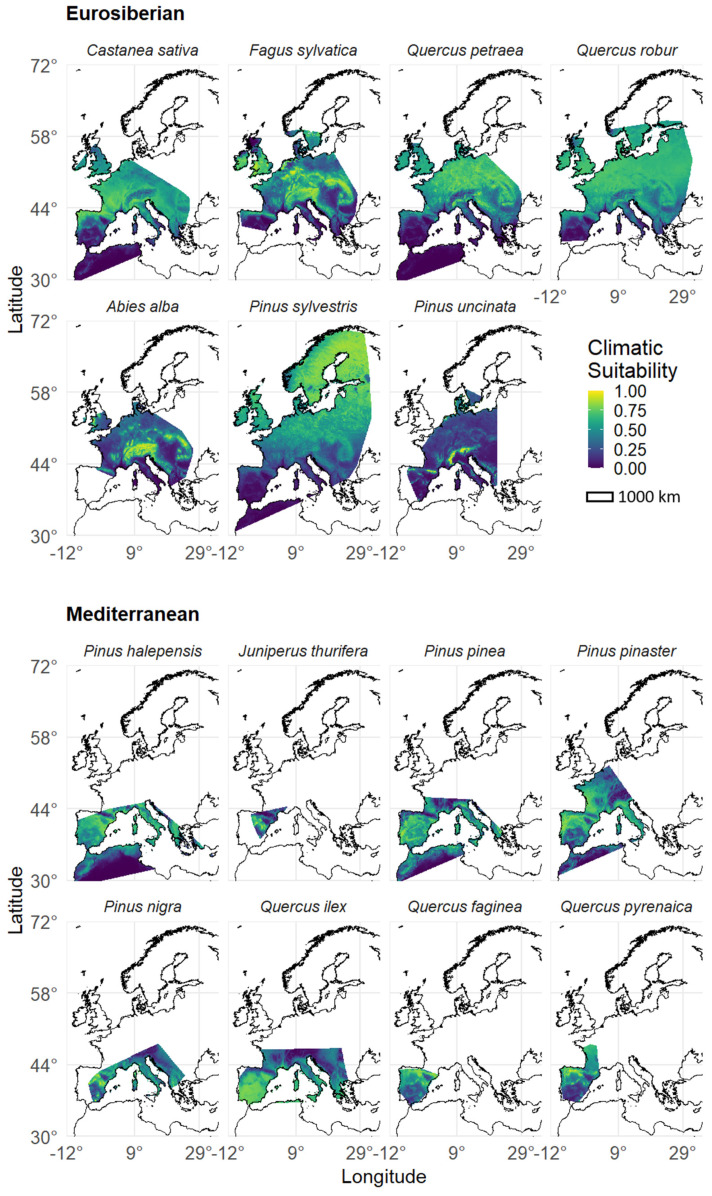
Spatial distribution of climatic suitability derived from MaxEnt models for the studied species across entire European range. Maps represent mean annual climatic suitability over the study period (1981–2005).

Geographic projections reflected these ecological differences (Figure 3). Eurosiberian species had highest suitability in Central and Northern Europe, with fragmented distributions in northern Iberian mountains at their southern range edges. *P. uncinata* was highly restricted to the Pyrenees. Mediterranean species peaked in the circum-Mediterranean region and the Iberian Peninsula, particularly along coasts and southern lowlands, while some taxa (e.g., *J. thurifera*, *P. nigra*) were concentrated in central plateaus and mountain systems. Transitional species like *Q. pyrenaica* and *Q. faginea* showed intermediate suitability, forming corridors between temperate and Mediterranean climates. Overall, the Iberian Peninsula emerged as a highly heterogeneous region, where suitability gradients of both groups overlap.

### 3.2. Climatic Suitability–Growth Coupling

LMM revealed a significant association between growth reduction and spatial gradients of climatic suitability across the Iberian Peninsula (Table 1). Mean climatic suitability emerged as a strong predictor of growth reduction, showing a significant negative effect indicating lower growth reduction values at higher suitability levels. When accounting for the temporal dimension of climatic suitability, both its mean and temporal variability contributed significantly to explaining growth reduction patterns. Mean suitability retained a negative effect, while suitability variability also showed a significant negative association with growth reduction. Model comparison supported the inclusion of the variability term, with a significant likelihood ratio test (χ^2^ = 6.34, df = 1, *p* = 0.011) and a reduction in AIC, indicating improved model fit. The LMM analysis revealed significant differences in growth reduction levels between biogeographic groups (Table 1). Specifically, Mediterranean species showed higher mean growth reduction values compared to Eurosiberian species across the study period.

## 4. Discussion

### 4.1. Forest Climatic Suitability Derived from SDMs

SDMs showed consistently robust performance across the 15 analyzed tree species, indicating that the main climatic gradients structuring European forest distributions were effectively captured [47,48]. Across species, climatic predictor importance and response curve shapes were strongly structured by biogeographic affiliation, revealing two contrasting niche architectures consistent with European forest biogeography [48,49]. Eurosiberian species were characterized by the dominant influence of evapotranspiration and maximum temperature, with narrow, unimodal optima centered on cool and humid conditions. This pattern reflects strong niche conservatism and limited tolerance to increased evaporative demand, in line with the documented sensitivity of temperate and montane forests to drought and warming at their southern range margins [24,50,51]. In contrast, Mediterranean species exhibited broader response curves and more heterogeneous contributions of climatic predictors, indicative of wider climatic tolerance and greater interannual variability in niche occupancy. These patterns are consistent with an evolutionary history shaped by seasonal drought and climatic unpredictability, favoring diversified and flexible adaptive strategies [52,53,54]. Compared to Eurosiberian species, Mediterranean species displayed less constrained niche architectures, reflecting contrasting ecological strategies rather than simple shifts along the same climatic axes [55].

Spatial projections further reinforced this biogeographic differentiation. Eurosiberian species showed suitability maxima concentrated in central and northern Europe, with suitability in southern Europe largely restricted to major mountain systems, highlighting the role of orography as a source of climatic buffering and potential refugia under warming scenarios [48,56,57]. Mediterranean species, by contrast, exhibited cores of high suitability in southern and southwestern Europe, with sharp declines toward higher latitudes, consistent with strong thermal and photoperiodic constraints beyond the Mediterranean climatic domain [58,59,60]. Overall, the limited spatial overlap between high-suitability areas of Eurosiberian and Mediterranean species underscores the Iberian Peninsula—particularly its mountainous and transitional regions—as a zone of pronounced climatic turnover. These ecotonal areas likely represent hotspots of climate sensitivity, where ongoing warming may disproportionately amplify growth limitations and forest decline processes, as previously reported for climatically marginal forest systems [14,61]. Taken together, these results confirm that SDM-derived climatic suitability provides a robust biogeographic and functional framework for identifying regions where climatic constraints on forest performance are likely to intensify under future climate change.

### 4.2. Low Climatic Suitability and Variability Impacts on Forest Growth

Our results provide empirical evidence that climatic suitability gradients derived from SDMs are robustly coupled with tree growth of European forest species. The significant negative association found between mean annual suitability and growth reduction supports the fundamental, yet often contested, assumption that SDM-derived indices serve as effective proxies for individual-level performance [5]. While previous syntheses have occasionally reported weak correlations between suitability and demography [1,10], our findings align with recent evidence suggesting that when SDMs are calibrated at biologically relevant scales and coupled with high-resolution performance data, they effectively capture the physiological constraints governing population persistence [4,16,17,62].

The strength of this coupling in the Iberian Peninsula highlights the role of climatic filtering as a primary determinant of tree vigor at regional scales. By utilizing annually resolved suitability metrics, we demonstrate that the climatic suitability projected by MaxEnt is not merely a static geographic abstraction but a dynamic reflection of the environmental energy and water balance that limits cambial activity [63,64]. This is particularly evident in the dominant role of evapotranspiration across both Eurosiberian and Mediterranean group, confirming that water-energy dynamics are the main drivers of the ecological niche and, consequently, of the growth potential in southwestern Europe [21]. Furthermore, the consistency of this relationship across 15 species with divergent biogeographic affinities suggests that the climatic suitability–growth link is a generalized feature of European forests. This correspondence validates the use of SDMs to identify “vulnerability hotspots”—areas where low suitability values precede detectable shifts in range limits. As growth reduction is a sensitive precursor to tree mortality [15], the ability of SDMs to track these declines provides a critical tool for anticipating the erosion of forest resilience under climate change [16]. By transforming suitability from a descriptive distributional metric into a process-based indicator of performance, our study bridges the gap between macroecology and forest monitoring [18].

Contrary to expectations that climatic instability primarily amplifies forest vulnerability [65], a notable finding of our study is the counterintuitive association between climatic instability and forest performance. The significant negative coefficient for the standard deviation of suitability indicates that increased interannual variability in suitability is associated with lower levels of growth reduction. While this could be interpreted as a form of physiological acclimation or ‘climatic priming’ [66,67,68], where recurrent fluctuations prepare the tree for stress, this hypothesis remains tentative. Other non-exclusive processes, such as local adaptation or the selection of more resilient genotypes in fluctuating environments, could also explain this trend. Further research integrating functional traits and genetic data is needed to confirm the underlying mechanisms of this observed resilience.

The additive effect of biogeographic affinity revealed a consistent offset in performance between the two studied groups. With a positive coefficient for the Mediterranean species, our results indicate that, for any given level of climatic suitability, Mediterranean species exhibit significantly higher growth reduction than their Eurosiberian counterparts. This finding contradicts the intuitive expectation that Mediterranean taxa, being functionally adapted to aridity, would maintain greater stability in radial growth within their core distribution area [69]. Several non-exclusive mechanisms may explain this apparent vulnerability. First, Mediterranean forests are subject to a higher frequency and intensity of compound climatic extremes, such as heatwaves co-occurring with multi-year droughts, which can impose physiological costs that exceed the predictive capacity of mean suitability indices [21,22]. While Eurosiberian species in the Iberian Peninsula are often restricted to topographically buffered refugia, Mediterranean taxa are directly exposed to the intensifying summer stress, potentially keeping them in a persistent state close to their hydraulic thresholds [70,71]. Consequently, even within climatically suitable areas, Mediterranean species may experience more frequent and severe growth declines driven by extreme events [72,73], not captured by the mean annual suitability.

We acknowledge that climatic suitability, while a powerful predictor, does not operate in isolation. In many cases, the direct link between climate and radial growth can be decoupled by local-scale factors. For instance, edaphic properties, micro-topographic buffering, and groundwater availability can act as micro-refugia [4,7]. Furthermore, biotic interactions (e.g., competition or pest outbreaks) and historical forest management practices can introduce significant ‘noise’ into the dendrochronological signal [3,74]. While some of these drivers remain difficult to quantify at large scales, they represent inherent constraints to the predictive power of macro-climatic models. Therefore, our suitability metrics should be interpreted as a measure of climatic potential rather than a strictly deterministic demographic outcome. Regarding our statistical approach, we utilized LMMs to quantify the coupling between suitability and growth due to their robustness in handling hierarchical data structures. However, we acknowledge that ecological responses are often non-linear. Future research could explore more flexible frameworks, such as Generalized Additive Models or threshold-based regressions, which might better capture potential non-linearities or ‘tipping points’ in forest responses to climatic suitability fluctuations [75].

### 4.3. Conservation Implications and Concluding Remarks

The integration of SDM-derived climatic suitability with dendrochronological data provides a functional bridge between macroecological patterns and local demographic processes. Our findings emphasize that climatic suitability index should be transitioned from a static mapping tool to a dynamic indicator of physiological stress [18]. This shift is critical for forest management under the current climate emergency, as it allows for the identification of vulnerability hotspots where low suitability and low variability converge to maximize growth reduction.

In the context of the Iberian Peninsula, our results suggest that conservation efforts should not only focus on the southern rear-edge limits of Eurosiberian species but also pay close attention to Mediterranean taxa in their core areas. The higher growth sensitivity of the Mediterranean group indicates that these species may be less resilient than previously assumed, operating dangerously close to their physiological limits [71]. For these populations, management strategies such as adaptive thinning to reduce competition for water or the promotion of structural heterogeneity may be essential to buffer against the increasingly frequent extreme events [74].

Our findings regarding the resilience of populations in variable environments suggest potential avenues for forest management. For example, populations from high-variability areas could be candidates for assisted gene flow [76,77,78]. However, we emphasize that these implications are currently exploratory. Any management decision involving the movement of genetic material must be supported by further site-specific trials that account for local adaptation to non-climatic factors, such as soil chemistry and photoperiod, which were not evaluated in this study.

Our results also contribute to the ongoing debate regarding the poleward migration of European forests under climate change [79]. While a northward shift in climatic suitability is expected, the actual migration of tree species is often lagged by dispersal limitations and the persistence of established individuals at their southern limits [80]. The strong sensitivity to annual suitability found in Mediterranean taxa suggests that these ‘rear-edge’ populations may act as early-warning systems; however, their ability to maintain stable growth levels during fluctuations indicates a physiological resilience that could buffer the immediate necessity of latitudinal shifts [56]. Understanding this suitability–growth coupling is therefore essential for predicting whether species will effectively track their climatic niche or form ‘extinction debts’ in their current ranges.

## Figures and Tables

**Table 1:** Estimated coefficients (±standard error) for the linear mixed-effects models explaining growth reduction (rw) as a function of climatic suitability and biogeographical affinity variables. Significance: ^ *p* < 0.10, ** *p* < 0.01, * *p* < 0.05, *** *p* < 0.001.

Model	Intercept	Suit_Inter	Suit_SD	Distrib	AICc
rw ~ suit_inter + distrib	0.075 ± 0.028 *	−1.520 ± 0.385 ***	-	0.180 ± 0.037 ***	−715.3
rw ~ suit_inter + suit_SD + distrib	0.076 ± 0.027 *	−1.637 ± 0.387 ***	−0.6711 ± 0.264 ***	0.183 ± 0.036 ***	−719.6

## Data Availability

All datasets were downloaded from public sources and previously published articles. See Section 2.

## Appendix A

**Table A1:** List of the tree species considered for this study. For each species, the scientific name is accompanied with the main region of the species (MED, Mediterranean; EU, Eurosiberian), the number of sites with available chronologies in the period 1981–2005 and the mean of reduction growth.

Species	Region	No. Sites (No. Trees)	Growth Reduction (Mean)
*Abies alba*	EU	5 (25)	0.02
*Castanea sativa*	EU	8 (40)	0.103
*Fagus sylvatica*	EU	25 (125)	0.143
*Pinus uncinata*	EU	19 (95)	0.04
*Pinus sylvestris*	EU	49 (245)	0.134
*Quercus petraea*	EU	3 (15)	0.117
*Quercus robur*	EU	2 (10)	0.106
*Juniperus thurifera*	MED	12 (60)	0.146
*Pinus halepensis*	MED	73 (365)	0.344
*Pinus nigra*	MED	41 (205)	0.243
*Pinus pinaster*	MED	18 (90)	0.286
*Pinus pinea*	MED	9 (45)	0.388
*Quercus faginea*	MED	17 (85)	0.158
*Quercus ilex*	MED	15 (75)	0.216
*Quercus pyrenaica*	MED	26 (130)	0.147

**Table A2:** Predictive performance of the Species Distribution Models based on the Area Under the Curve (AUC) metric for the studied species.

Species	Region	AUC (Mean ± SD)
*Abies alba*	EU	0.803 ± 0.018
*Castanea sativa*	EU	0.786 ± 0.017
*Fagus sylvatica*	EU	0.714 ± 0.014
*Pinus uncinata*	EU	0.905 ± 0.013
*Pinus sylvestris*	EU	0.724 ± 0.011
*Quercus petraea*	EU	0.786 ± 0.016
*Quercus robur*	EU	0.664 ± 0.022
*Juniperus thurifera*	MED	0.821 ± 0.027
*Pinus halepensis*	MED	0.804 ± 0.02
*Pinus nigra*	MED	0.771 ± 0.043
*Pinus pinaster*	MED	0.756 ± 0.021
*Pinus pinea*	MED	0.752 ± 0.024
*Quercus faginea*	MED	0.701 ± 0.025
*Quercus ilex*	MED	0.714 ± 0.02
*Quercus pyrenaica*	MED	0.726 ± 0.018

**Table A3:** Relative importance of climatic predictors in MaxEnt models for each study species. Values are expressed as the mean contribution of each variable across the study period (1981–2005) and interannual standard deviation (SD), reflecting temporal variability in variable importance.

Region	Species	Variable	Importance (Mean ± SD)
EU	*Abies alba*	evapotranspiration	37.376 ± 12.399
EU		maximum temperature	0.786 ± 1.447
EU		precipitation	61.845 ± 11.688
EU	*Castanea sativa*	evapotranspiration	67.459 ± 17.819
EU		maximum temperature	10.5 ± 7.485
EU		precipitation	22.041 ± 17.677
EU	*Fagus sylvatica*	evapotranspiration	21.89 ± 9.375
EU		maximum temperature	54.085 ± 10.573
EU		precipitation	24.032 ± 6.693
EU	*Quercus petraea*	evapotranspiration	80.448 ± 5.211
EU		maximum temperature	13.897 ± 3.855
EU		precipitation	5.652 ± 5.186
EU	*Quercus robur*	evapotranspiration	71.783 ± 14.902
EU		maximum temperature	13.9 ± 9.893
EU		precipitation	14.31 ± 12.452
EU	*Pinus sylvestris*	evapotranspiration	91.569 ± 2.625
EU		maximum temperature	5.414 ± 3.141
EU		precipitation	3.017 ± 2.562
EU	*Pinus uncinata*	evapotranspiration	6.838 ± 3.674
EU		maximum temperature	90.372 ± 6.939
EU		precipitation	2.793 ± 4.869
MED	*Pinus halepensis*	evapotranspiration	61.845 ± 14.287
MED		maximum temperature	3.631 ± 5.069
MED		precipitation	34.514 ± 14.268
MED	*Juniperus thurifera*	evapotranspiration	27.541 ± 13.634
MED		maximum temperature	29.993 ± 9.075
MED		precipitation	42.448 ± 16.151
MED	*Pinus pinea*	evapotranspiration	50.307 ± 11.232
MED		maximum temperature	13.821 ± 9.301
MED		precipitation	35.866 ± 14.808
MED	*Pinus pinaster*	evapotranspiration	71.945 ± 11.661
MED		maximum temperature	6.941 ± 6.223
MED		precipitation	21.117 ± 11.051
MED	*Pinus nigra*	evapotranspiration	39.296 ± 16.121
MED		maximum temperature	29.572 ± 10.667
MED		precipitation	31.144 ± 18.508
MED	*Quercus ilex*	evapotranspiration	48.793 ± 30.056
MED		maximum temperature	6.952 ± 11.863
MED		precipitation	44.252 ± 32.463
MED	*Quercus faginea*	evapotranspiration	57.152 ± 24.704
MED		maximum temperature	7.414 ± 10.713
MED		precipitation	35.445 ± 23.866
MED	*Quercus pyrenaica*	evapotranspiration	23.804 ± 17.942
MED		maximum temperature	38.171 ± 18.436
		precipitation	38.029 ± 22.384
